# Venous and arterial cerebral thrombosis: a COVID-19 dual pathology and single possible etiology—a case report

**DOI:** 10.1186/s41983-021-00443-9

**Published:** 2022-01-15

**Authors:** Tamer Roushdy, Nouran K. Sharaf

**Affiliations:** Neurology Department, Faculty of Medicine, Ain Shams University, 38 Abbasia, Cairo, 11591 Egypt

**Keywords:** COVID-19, Sinus thrombosis, Border zone infarcts, Venous and arterial thrombosis, Covid induced coagulopathy

## Abstract

**Background:**

Corona virus disease of the year 2019 (COVID-19) is still devastating the world for more than 19 months since its declaration as a pandemic by world health organization. Its manifestations does not stand at respiratory system but involves other body systems including central nervous system and its vasculature. In the following case report, cerebral venous and arterial thrombosis is detected in a case just in convalescence from COVID-19 with still detected positive IgM.

**Case presentation:**

A 68-year-old female presenting with disturbed conscious level, bilateral convergent squint, single attack of generalized seizures, left sided dense weakness within a short time from catching COVID-19 and while still in quarantine hospital in recovery phase from infection. Magnetic resonance studies revealed bilateral cortical border zone infarcts as well as left lateral dural sinus and deep venous thrombosis.

**Conclusion:**

Along the forth wave, COVID-19 is still hitting hardly the central nervous system vasculature.

## Background

Novel severe acute respiratory syndrome Corona virus 2 (SARS-CoV-2) has been associated with liability for coagulopathy and immunothrombosis through different means [1]. Plenty of reports discussing COVID-19 and strokes whether arterial or venous are published along more than 17 months since its pandemic declaration [2, 3].

To our knowledge dual pathology being venous and arterial thrombosis with a single common etiology that is COVID-19 in a single case is not commonly reported.

## Case presentation

A 68-year-old female, compliant and controlled on her medications for hypertension, diabetes, and with paroxysmal atrial fibrillation (AF) on new oral anticoagulants with a controlled rate diagnosed positive for COVID-19 on clinical basis as well as through polymerase chain reaction (PCR) in September 2021. Patient was admitted to quarantine hospital with an oxygen saturation of 90% and discharged home after 20 days. Few days later the patient began to complain of severe throbbing headache radiating to the back of head and increasing on lying flat that was followed by a single unprovoked generalized tonic clonic seizure followed by disturbed conscious level with a Glasgow coma scale (GCS) of 10/15. Patient was readmitted to an intensive care unit and on retesting PCR it was still positive.

**Fig. 1:**
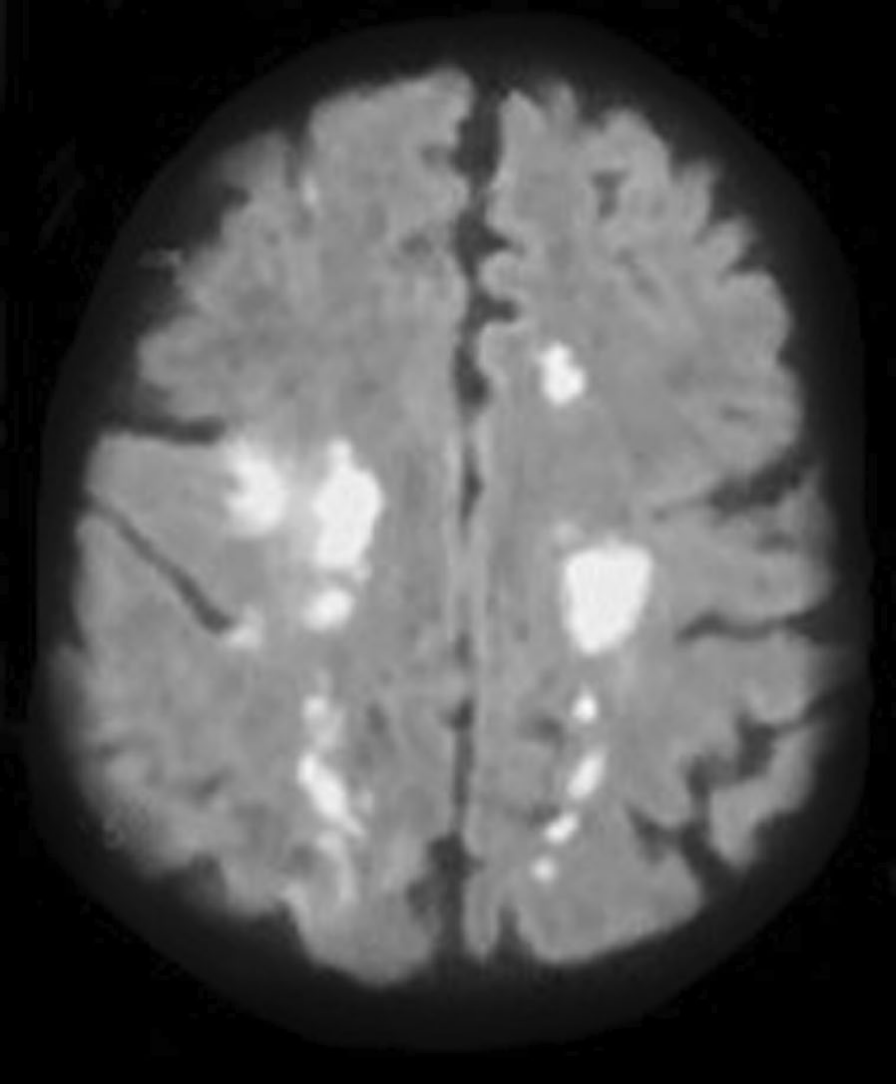
Bilateral cortical border zone (watershed) infarcts as evident by restricted diffusion (DWI)

**Fig. 2:**
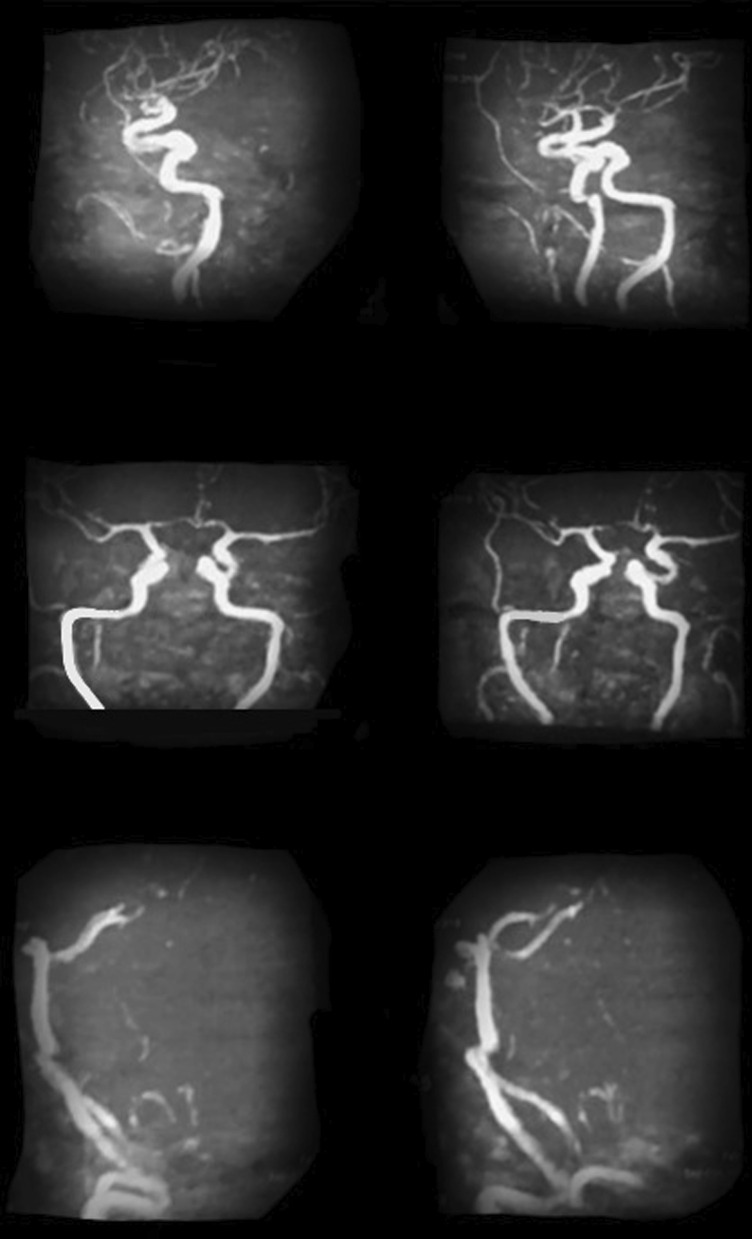
Magnetic resonance angiography revealing diffuse atherosclerosis and hypo plastic A1 segment of anterior cerebral artery (normal variant) with no stenosis or hemodynamic instability

**Fig. 3:**
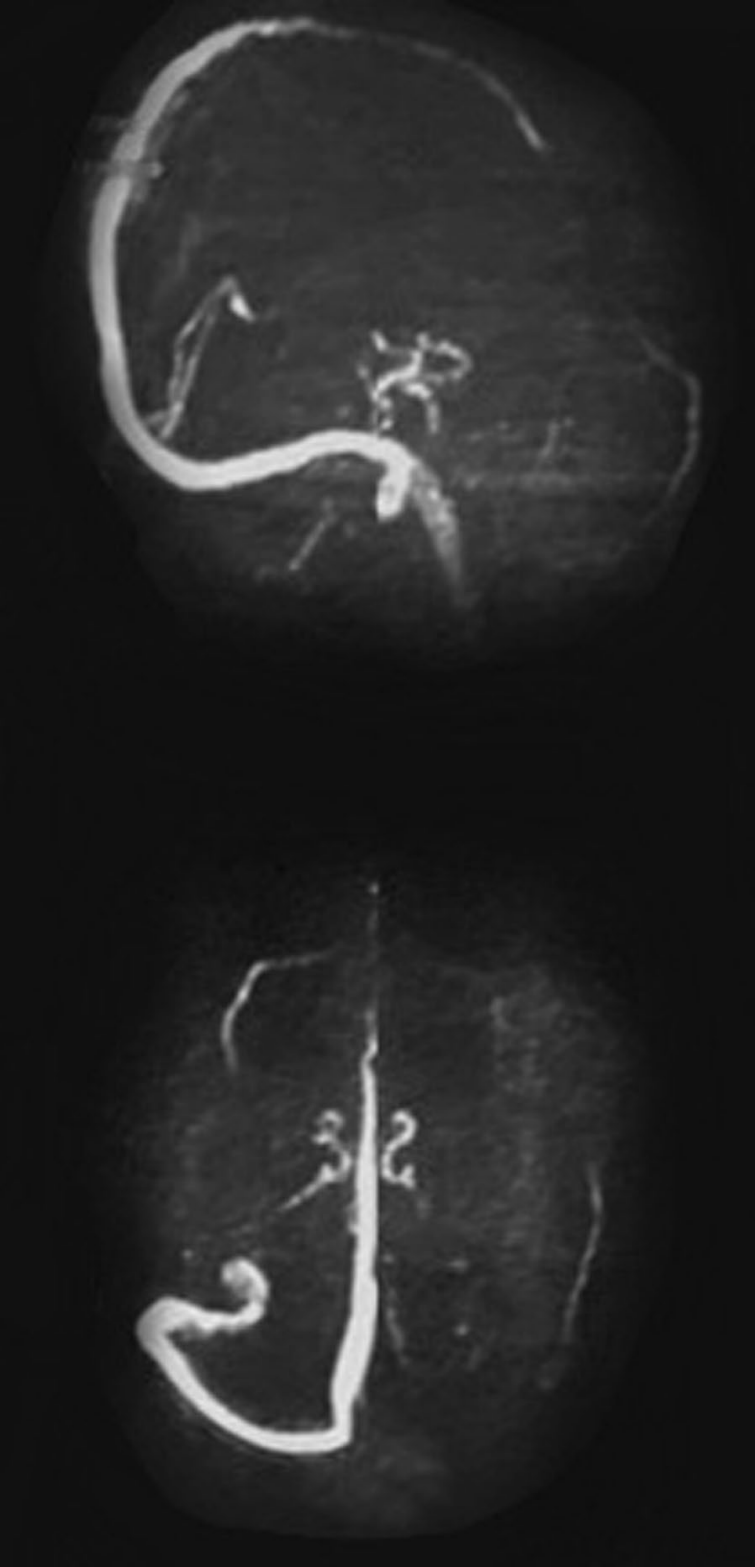
Left lateral dural venous sinuses thrombosis and deep vein thrombosis as evident through MRV

Ten days later the patient was discharged yet with a bilateral convergent squint more on her right eye and left sided weakness with national institute of health stroke scale (NIHSS) of 12. Patient sought medical advice at emergency room (ER) and was admitted to stroke center, where magnetic resonance imaging (MRI) (Philips 1.5 Tesla, Germany) stroke protocol was done as well as magnetic resonance venography (MRV) that revealed bilateral cortical border zone infarcts with restricted diffusion along DWI (Fig. 1) with diffuse atherosclerosis and mural irregularity, attenuated beaded lumen and hypo plastic left anterior cerebral artery A1 segment (normal variant) on magnetic resonance angiography (MRA) (Fig. 2) and altered signal of left lateral dural venous sinuses as well as deep venous system thrombosis with no flow related signal in MRV (Fig. 3).

Basic laboratory samples were ordered and results were going with mild elevation of Prothrombin time / activated Partial thromboplastin time (PT / aPPT) 12.3/23.2 (normal range 9.8–12.1/26.4–37.5), D-dimer 0.95, ferritin 726.2, CRP 9.03, low normal platelet count 160.000, relative lymphopenia 14.9% (normal range 20–40%).

Laboratory blood samples for collagen disorders and thrombophilia were obtained including anti-thrombin III, Protein C and S, factor V Leiden, ANA, anti DNA, anti-cardiolipin antibody and lupus anticoagulant and all were within normal limits, beside absence of clinical history suggestive of collagen disorders or previous history of thrombosis.

Neurosonology with color coded duplex study of both extracranial carotid tree and vertebral system and Doppler spectral wave analysis as well as transcranial duplex (Acuson Juniper, Siemens, Seoul) revealed diffuse atherosclerosis with no evidence of hemodynamic changes or stenosis.

Electrocardiogram (ECG) (CM 300A, Comen, China) revealed normal sinus rhythm and long term monitoring revealed controlled rate paroxysmal AF. Transthoracic echocardiography (Vivid E9 machine, General Electric, Vingmed Ultrasound, Horten, Norway) was within accepted limits with ejection fraction 76% and left atrium diameter of 32 with no evidence of intracardiac thrombus or wall abnormality.

Patient was tested for COVID-19 by PCR that was negative on two successive tests within a 48 h interval as for IgG it was positive donating previous infection and IgM was weak positive donating convalescence phase.

**Fig. 4:**
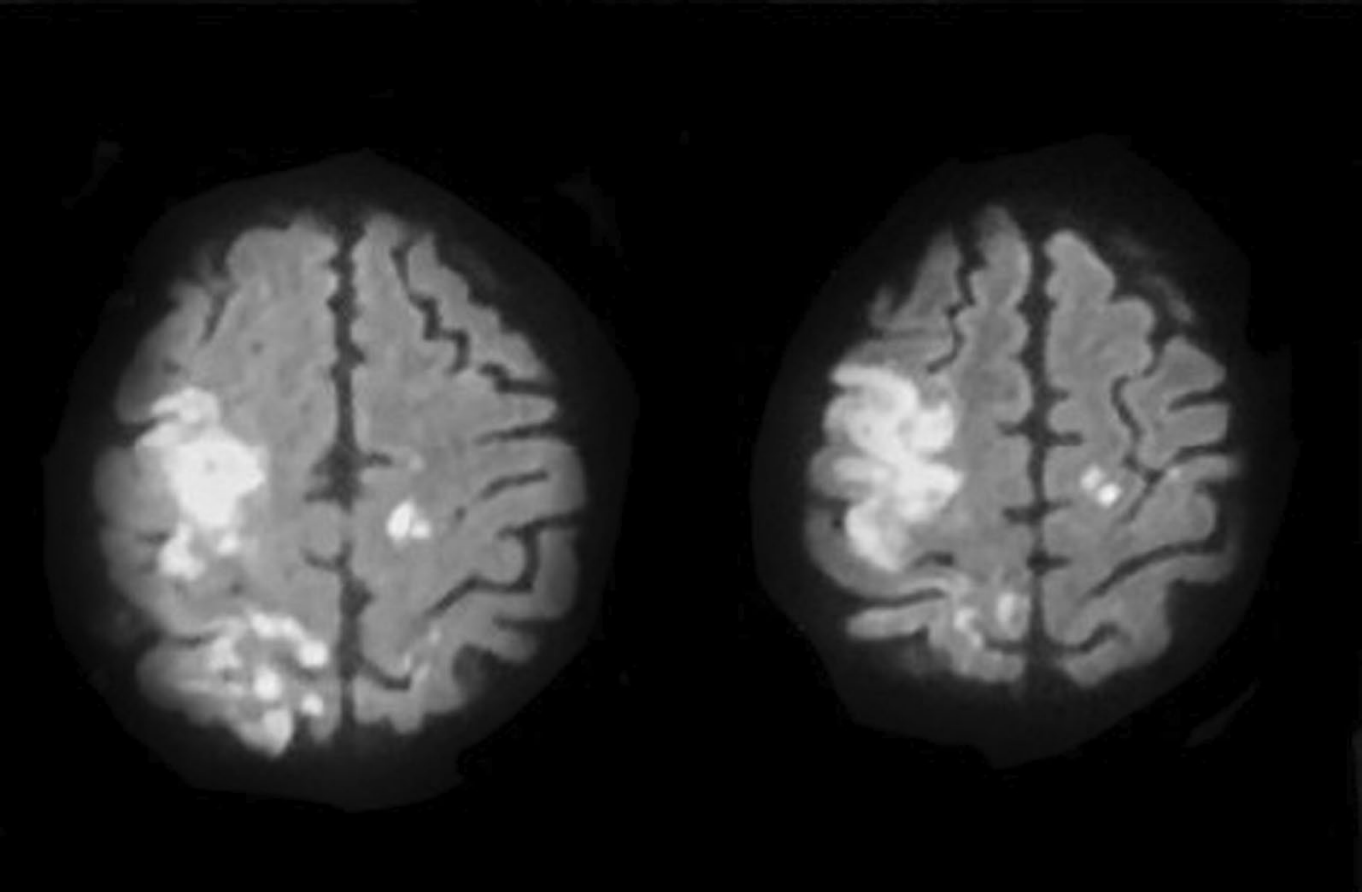
Moderate-sized right high frontal cortical and sub-cortical lesion with heterogeneous signal and partly diffusion restriction going with venous infarction

Patient was placed on full dose anticoagulation beside antiepileptic and dehydrating measures with initial improvement regarding sensorium yet recurrent deterioration took place with GCS 6. Follow-up MRI revealed cortical venous infarction donating unresponsive venous system thrombosis to treatment (Fig. 4). Patient was intubated and ventilated and unfortunately died 3 days later.

A final diagnosis was that the patient had dual arterial as well as venous thrombosis, venous thrombosis had no other possible explanation rather than COVID-19 induced hyper coagulopathy (CIC), while arterial thrombosis was either COVID-19 associated or secondary to the patient medical past history.

## Discussion

Severe acute respiratory syndrome Corona virus 2 is no longer considered a novel virus after bombarding the globe for more than 22 months since December 2019 when it was first noticed in Wuhan province in China. Meanwhile, its novel manifestations are still going on.

Along the current case report a dual pathology of venous and arterial thrombosis is diagnosed in a 68-year-old female patient that was just recovering from COVID-19.

Accounting for 0.5% of all strokes; dural vein thrombosis is considered a rare etiological cause of stroke [4]. Rough estimates states that it can affect 3 to 4 individuals per million. Venous thrombosis whether dural or deep that was clinically and radiologically diagnosed in this case cannot have another possible explanation except CIC taking into consideration the baseline laboratory results that were having an elevated inflammatory markers, elevated D-dimer, relative increase of PT/PTT ratio that are considered laboratory criteria for CIC [5–7] and normal laboratory results for collagen disorders or thrombophilia.

Severe acute respiratory syndrome Corona virus 2 enters the body through receptors on endothelial cells causing damage in the endothelial cell wall with no longer properly functioning endothelial cells either as a smooth lining for blood vessels or as a source of pro or anticoagulant factors that either stabilize clot or reduce excessive coagulation [1].

Virchow’s triad that was described in the nineteenth century which is composed of hyper coagulopathy, endothelial damage, and blood stasis is resurrected again with COVID-19 and its associated thrombosis [8].

As for arterial border zone infarcts that were detected within the presented case, although having history of vascular risk factors being hypertensive and diabetic yet the patient’s vitals and laboratory results were within accepted ranges all through the hospitalization period and she was complaint and controlled on her medications.

Also vascular investigations that can reveal stenosis that may have a role in border zone infarcts development as MRA and Neurosonology did not reveal any significant abnormality other than diffuse atherosclerosis.

As for AF which is a possible explanation for bilateral cortical watershed infarcts that accounts for 5–10% of all strokes [9]. Atrial fibrillation is either paroxysmal or non-paroxysmal and according to varies studies risk of thromboembolism is less in paroxysmal than non-paroxysmal ones [10, 11]. The presented case had a history of paroxysmal controlled AF and was on anticoagulation which can at least partially exempt AF from being responsible for such arterial infarcts and again accuse COVID-19 for such infarcts.

## Conclusion

Corona virus disease of the year 2019 is still presenting with novel manifestations along the forth wave. Two forms of strokes being dural and deep venous thrombosis as well as cortical border zone arterial infarcts are detected in a single case just in the convalescence phase of COVID-19.

## Data Availability

The corresponding author takes full responsibility for the data, has full access to all of the data, and has the right to publish any and all data separate and apart from any sponsor.

## Abbreviations

COVID-19: Corona virus disease of the year 2019
SARS-CoV-2: Severe acute respiratory syndrome Corona virus 2
AF: Atrial fibrillation
PCR: Polymerase chain reaction
GCS: Glasgow coma scale
NIHSS: National institute of health stroke scale
ER: Emergency room
MRI: Magnetic resonance imaging
MRV: Magnetic resonance venography
PT: Prothrombin time
aPPT: Activated partial thromboplastin time
ECG: Electrocardiogram
CIC: COVID-19-induced hypercoagulopathy
